# The Fatty Acid β-Oxidation Pathway is Activated by Leucine Deprivation in HepG2 Cells: A Comparative Proteomics Study

**DOI:** 10.1038/s41598-017-02131-2

**Published:** 2017-05-15

**Authors:** Guokai Yan, Xiuzhi Li, Ying Peng, Baisheng Long, Qiwen Fan, Zhichang Wang, Min Shi, Chunlin Xie, Li Zhao, Xianghua Yan

**Affiliations:** 1 College of Animal Sciences and Technology, Huazhong Agricultural University, Wuhan, 430070 Hubei China; 2 The Cooperative Innovation Center for Sustainable Pig Production, Wuhan, 430070 Hubei China; 3 Hubei Provincial Engineering Laboratory for Pig Precision Feeding and Feed Safety, Wuhan, 430070 Hubei China; 4 State Key Laboratory of Agricultural Microbiology, College of Veterinary Medicine, Huazhong Agricultural University, Wuhan, 430070 Hubei China

## Abstract

Leucine (Leu) is a multifunctional essential amino acid that plays crucial role in various cellular processes. However, the integral effect of Leu on the hepatic proteome remains largely unknown. Here, we for the first time applied an isobaric tags for relative and absolute quantification (iTRAQ)-based comparative proteomics strategy to investigate the proteome alteration induced by Leu deprivation in human hepatocellular carcinoma (HepG2) cells. A total of 4,111 proteins were quantified; 43 proteins were further identified as differentially expressed proteins between the normal and Leu deprivation groups. Bioinformatics analysis showed that the differentially expressed proteins were involved in various metabolic processes, including amino acid and lipid metabolism, as well as degradation of ethanol. Interestingly, several proteins involved in the fatty acid β-oxidation pathway, including ACSL1, ACADS, and ACOX1, were up-regulated by Leu deprivation. In addition, Leu deprivation led to the reduction of cellular triglycerides in HepG2 cells. These results reveal that the fatty acid β-oxidation pathway is activated by Leu deprivation in HepG2 cells, and provide new insights into the regulatory function of Leu in multiple cellular processes, especially fatty acid metabolism.

## Introduction

Leucine (Leu) is an essential amino acid that serves as one of the basic substrates for the synthesis of proteins and polypeptides. In addition, accumulating evidence has shown that Leu plays crucial signaling roles in regulating a wide range of physiological and pathological processes such as satiety^1, 2^, autophagy^3, 4^, energy homeostasis and insulin secretion^5^. Moreover, as a vital nutrient, Leu and its metabolites also affect the metabolism of other nutrients including carbohydrate, protein (amino acids), and lipid in various types of cells including hepatocytes^6^. Previously, a number of studies have demonstrated that Leu reduces lipid accumulation by promoting the oxidation of Leu in either myocytes or adipocytes^7, 8^. In isolated hepatocytes, however, the case may be different mainly due to the absence of the key enzyme mitochondrial branched-chain amino acid transaminase that catalyzes the first step for the Leu oxidation pathway^6, 9^. It has been observed that the triglyceride content increases with Leu supplementation in both hepatocytes and hepatoma carcinoma cells^10, 11^. However, the mechanisms underlying the role of Leu in regulating hepatic lipid metabolism are poorly understood.

Over the past years, several 2-dimensional gel electrophoresis (2-DE) based comparative proteomics strategies have been applied into the functional exploration of specific amino acids including arginine (Arg)^12^, glutamine (Gln)^13, 14^ and methionine (Met)^15^. For instance, coupling 2-DE with MALDI-TOF-MS, Lenaerts and co-workers^12^ studied the proteome characters of preconfluent or postconfluent Caco-2 cells treated with Arg deficiency and they found that several crucial proteins, such as the cellular apoptosis susceptibility proteins CAS and HSF, and cell proliferation related protein SET, were differentially regulated by Arg deprivation, thus providing a molecular insight into the effects of Arg on cellular proliferation and apoptosis. Deniel *et al*.^14^ investigated the effect of Gln on the proteome of human epithelial intestinal cell line HCT-8 dealing with apoptotic conditions, and they found that Gln altered the expressions of crucial apoptotic process related proteins including MAP3K7, Aven, Atg5, and stathmin, thus giving multiple hypotheses regarding to the anti-apoptosis capacities of Gln. Xin and colleagues^15^ investigated the effects of Met restriction on the proteome of gastric cancer cells SGC7901 by culturing cells in normal and Met-deprived homocysteine-supplemented media. They identified a total of ten proteins, which are related to cell cycle arrest and apoptosis, that were differentially regulated by Met restriction. These findings give us a more comprehensive understanding of the functionality of specific amino acids. However, due to the limited identification capacity of 2-DE based proteomics strategies^16^, the integral effect of specific amino acids on certain cells remains largely unknown.

**Figure 1:**
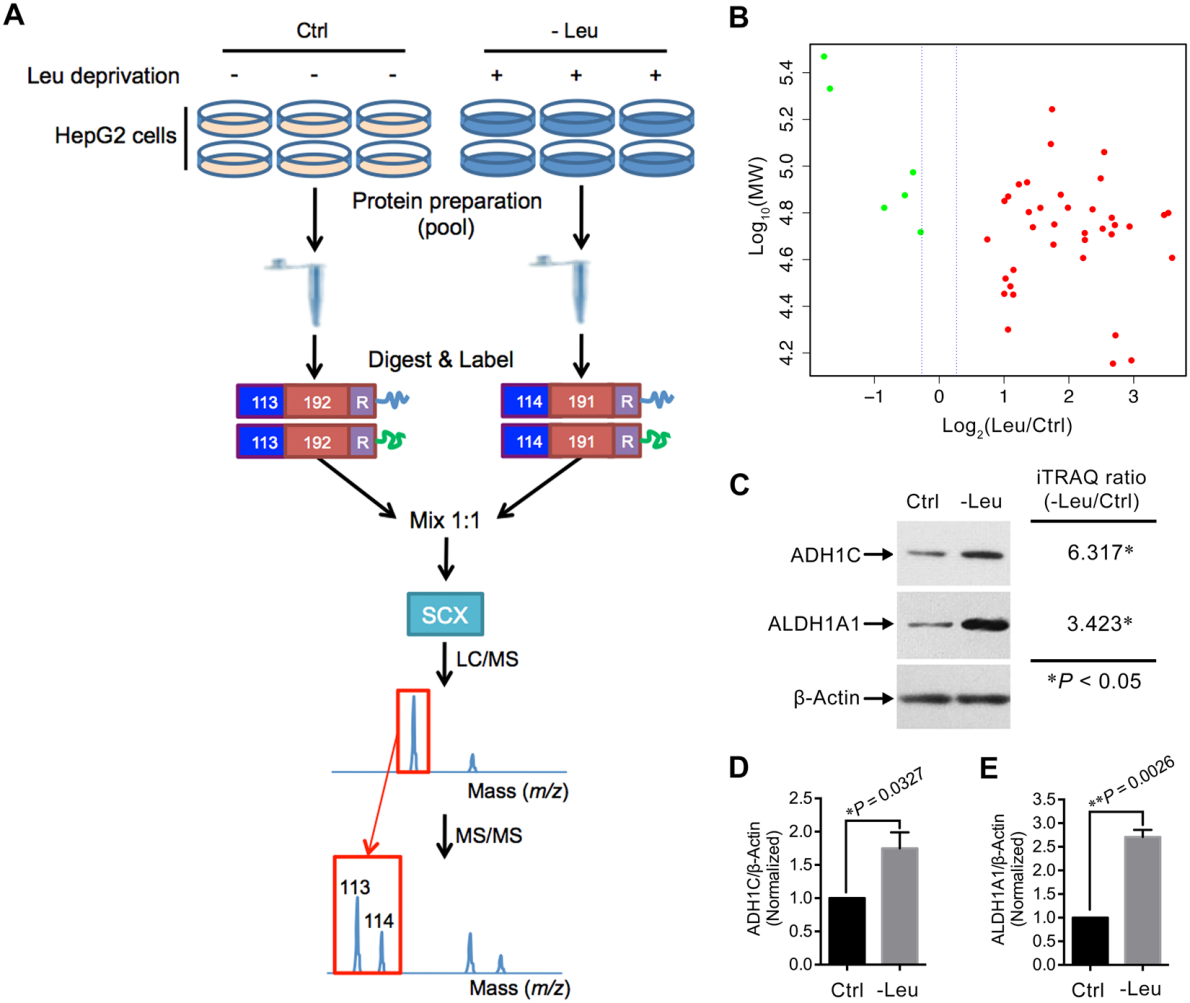
Identification of the differentially expressed proteins by the iTRAQ-based strategy. (**A**) Schematic diagram of the workflow for the iTRAQ-based experiments. Six dishes of HepG2 cells were pooled into one sample for either normal (Ctrl) or Leu-deprived (-Leu) HepG2 cells. Then pooled cells were fractionated, digested into peptides, labeled with different iTRAQ reagents containing reporter groups of different masses (113, 114), balance groups of different masses (192, 191), and a reactive group (R). The labeled peptides were then mixed equivalently, fractionated by strong cation exchange (SCX), and analyzed by LC-MS/MS. Two independent biological replicates were performed for the iTRAQ experiments. (**B**) The protein distribution in accordance to Log_10_ theoretical weight (MW; Da) versus log_2_ Ratio of all the differentially expressed proteins in -Leu/Ctrl. Red dots indicate up-regulated proteins, green dots indicate down-regulated. (**C**) Validation of the differential expressions of ADH1C and ALDH1A1 by immunoblotting. (**D,E**) Quantitation of ADH1C/β-Actin and ALDH1A1/β-Actin as described in (**C**). Data are means ± SD (n = 3). ^*^ *P* < 0.05, ^**^ *P* < 0.01 (paired Student’s *t*-test).

Since its development in 2004, the isobaric tags for relative and absolute quantification (iTRAQ) technique has become a powerful tool in comparative proteomics field due to several unique advantages including high-throughput, high sensitivity, and great accuracy^17, 18^. Previously, using the iTRAQ-based approach, we have characterized the proteome of HepG2 cells treated with either Gln deprivation or Arg supplementation^19, 20^. However, to our knowledge, there is no literature reporting proteome scale alterations induced by Leu deprivation in liver cells. Here, we conducted an iTRAQ-based proteomics study to profile the proteome of Leu-deprived HepG2 cells (Fig. 1A). Our bioinformatics analysis suggested that Leu deprivation regulated the expressions of proteins that are involved in several crucial biological processes such as amino acid metabolism and lipid metabolism. Simultaneously, our pathway analysis together with the validation assays indicated that the fatty acid β-oxidation pathway was significantly activated by Leu deprivation in HepG2 cells. The present study suggests a potential regulatory role of Leu on fatty acid metabolism in HepG2 cells.

## Results

### Quantitative proteomic analysis of HepG2 cells treated with Leu deprivation

By the iTRAQ-coupled LC-MS/MS analysis, a total of 4,111 proteins were quantified between Ctrl and -Leu in the two sets of biological replicates (Supplementary Fig. S1). Using a stringent criteria of unique peptide >1, *P*-value < 0.05, fold change >1.2 (or <0.833) in both the two sets of iTRAQ experiments, we identified 5 differentially expressed proteins for the -Leu/Ctrl (Table 1). In addition, due to the large variation between different iTRAQ runs (Supplementary Fig. S2), and to identify more proteins that were potentially regulated by Leu deprivation, we used a less stringent strategy with the criteria of unique peptide >1, *P*-value < 0.05, fold change >2 (or <0.5) in any one set of the two iTRAQ experiments (similar strategy was performed in refs 19, 21). By this strategy, 38 proteins were further identified as differentially expressed proteins (Table 1). Thus, there were a total of 43 proteins were finally considered as differentially expressed proteins for the -Leu/Ctrl (Table 1). Out of these proteins, 37 were up-regulated and 6 were down-regulated (Fig. 1B). In order to validate the differential expressions of proteins derived from the iTRAQ experiments, we immunoblotted two proteins alcohol dehydrogenase 1 C (ADH1C) and retinal dehydrogenase 1 (ALDH1A1; Fig. 1C). The selection of these two proteins was due to their potential correlation with the function of specific amino acid^19^, and the relative expressions of these proteins between Ctrl and -Leu groups were consistent with those determined by the iTRAQ approach (Fig. 1D,E).

**Table 1:** All the differentially expressed proteins identified by the iTRAQ experiments.

Accession	Description	Gene name	-Leu/Ctrl (Set1|Set2)	-Leu/Ctrl used for analysis
Proteins identified by the stringent criteria of unique peptide >1, *P*-value < 0.05, fold change >1.2 (or <0.833) in both the two sets of iTRAQ experiments				
Q86U79	Adenosine kinase	*ADK*	1.969*|1.376*	1.673
P57081	tRNA (guanine-N(7)-)-methyltransferase non-catalytic subunit WDR4	*WDR4*	0.830*|0.810*	0.820
Q9UNS1	Protein timeless homolog	*TIMELESS*	0.739*|0.773*	0.756
P04264	Keratin, type II cytoskeletal 1	*KRT1*	0.776*|0.609*	0.693
P13645	Keratin, type I cytoskeletal 10	*KRT10*	0.544*|0.567*	0.556
Proteins identified by the less stringent criteria of unique peptide >1, *P*-value < 0.05, fold change >2 (or <0.5) in any one set of the two iTRAQ experiments				
Q15493	Regucalcin	*RGN*	12.059*|-	12.059
Q9H2A2	Aldehyde dehydrogenase family 8 member A1	*ALDH8A1*	11.577*|-	11.577
P00439	Phenylalanine-4-hydroxylase	*PAH*	11.075*|-	11.075
A5YKK6	CCR4-NOT transcription complex subunit 1	*CNOT1*	7.806*|-	7.806
Q93088	Betaine–homocysteine S-methyltransferase 1	*BHMT*	7.652*|-	7.652
P52758	Ribonuclease UK114	*HRSP12*	6.583*|-	6.583
P05166	Propionyl-CoA carboxylase beta chain, mitochondrial	*PCCB*	6.541*|-	6.541
B3VL17	Beta globin (Fragment)	*HBB*	6.408*|-	6.408
Q3KPF3	Cytochrome P450, family 2, subfamily D, polypeptide 6	*CYP2D6*	6.326*|-	6.326
P00326	Alcohol dehydrogenase 1 C	*ADH1C*	6.317*|-	6.317
Q9UI17	Dimethylglycine dehydrogenase, mitochondrial	*DMGDH*	5.834*|-	5.834
O75367	Core histone macro-H2A.1	*H2AFY*	5.740*|-	5.740
P33121	Long-chain-fatty-acid–CoA ligase 1	*ACSL1*	5.621*|-	5.621
P30038	Delta-1-pyrroline-5-carboxylate dehydrogenase, mitochondrial	*ALDH4A1*	5.152*|-	5.152
P00480	Ornithine carbamoyltransferase, mitochondrial	*OTC*	4.747*|-	4.747
D4QEZ8	Short-chain acyl-CoA dehydrogenase	*ACADS*	4.739*|-	4.739
Q9Y365	PCTP-like protein	*STARD10*	4.659*|-	4.659
B2RBJ5	Alanine-glyoxylate aminotransferase 2 (AGXT2), nuclear gene encoding mitochondrial protein	*AGXT2*	3.959*|-	3.959
P83111	Serine beta-lactamase-like protein LACTB, mitochondrial	*LACTB*	3.667*|-	3.667
P00352	Retinal dehydrogenase 1	*ALDH1A1*	3.423*|-	3.423
P21695	Glycerol-3-phosphate dehydrogenase [NAD(+)], cytoplasmic	*GPD1*	3.393*|-	3.393
P47989	Xanthine dehydrogenase/oxidase	*XDH*	3.342*|-	3.342
Q3SY69	Mitochondrial 10-formyltetrahydrofolate dehydrogenase	*ALDH1L2*	3.299*|-	3.299
Q16696	Cytochrome P450 2A13	*CYP2A13*	2.950*|-	2.950
P26440	Isovaleryl-CoA dehydrogenase, mitochondrial	*IVD*	2.722*|-	2.722
Q3LXA3	Bifunctional ATP-dependent dihydroxyacetone kinase/FAD-AMP lyase (cyclizing)	*DAK*	2.606*|-	2.606
P42357	Histidine ammonia-lyase	*HAL*	2.559*|-	2.559
O14975	Very long-chain acyl-CoA synthetase	*SLC27A2*	2.343*|-	2.343
P07858	Cathepsin B	*CTSB*	2.212*|-	2.212
P62879	Guanine nucleotide-binding protein G(I)/G(S)/G(T) subunit beta-2	*GNB2*	2.207*|-	2.207
P09417	Dihydropteridine reductase	*QDPR*	2.140*|-	2.140
Q16134	Electron transfer flavoprotein-ubiquinone oxidoreductase, mitochondrial	*ETFDH*	2.090*|-	2.090
P09455	Retinol-binding protein 1	*RBP1*	2.087*|-	2.087
P60953	Cell division control protein 42 homolog	*CDC42*	2.032*|-	2.032
P32189	Glycerol kinase	*GK*	2.009*|-	2.009
P61916	Epididymal secretory protein E1	*NPC2*	2.005*|-	2.005
Q562E7	WD repeat-containing protein 81	*WDR81*	0.311*|-	0.311
Q8N201	Integrator complex subunit 1	*INTS1*	0.292*|-	0.292

^*^ *P* < 0.05.

### Subcellular and functional characterization of the differentially expressed proteins

**Figure 2:**
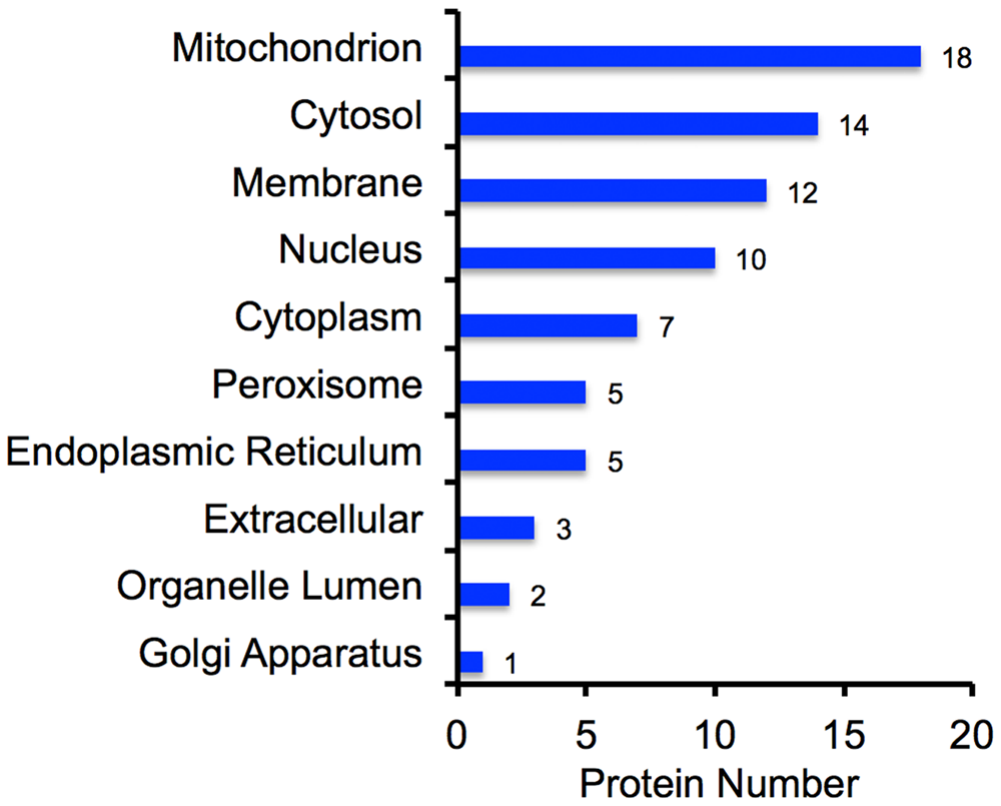
GO subcellular annotation for all the differentially expressed proteins in Leu-deprived HepG2 cells. Horizontal axis indicates the actual number of differentially expressed protein annotated in certain compartment.

We next catalogued the subcellular annotation of all the differentially expressed proteins. Based on the Gene Ontology (GO), the differentially expressed proteins were found to mainly localize to mitochondrion, cytosol, membrane, and nucleus (Fig. 2).

**Figure 3:**
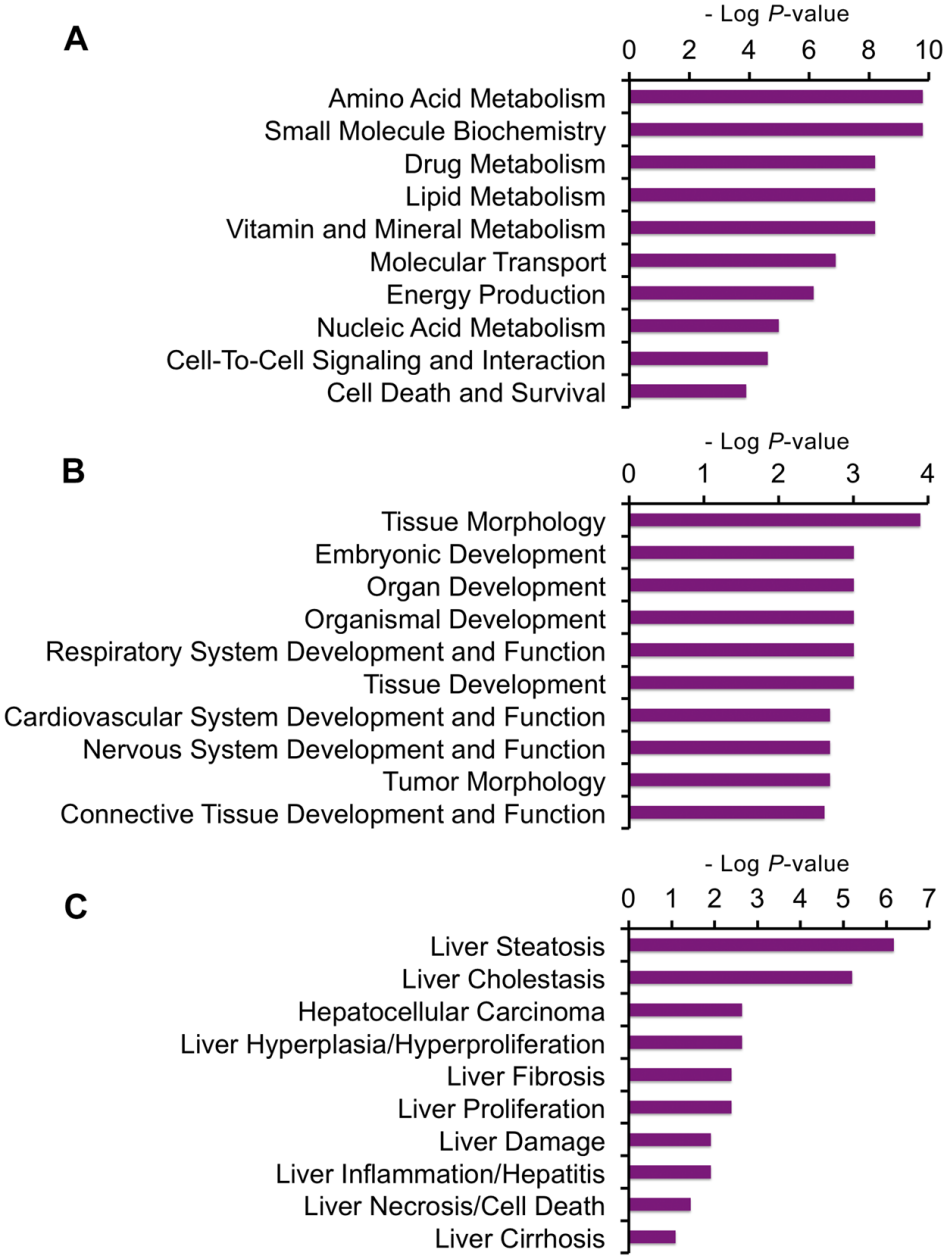
Functional characterization of the differentially expressed proteins in -Leu/Ctrl. (**A**) Molecular and cellular functions. (**B**) Physiological system development and functions. (**C**) Hepatotoxicity. More information is available in Supplementary Table S1.

To characterize the functional roles and biological processes of the differentially expressed proteins after the treatment of Leu deprivation, the protein Uniprot accessions and ratios of -Leu/Ctrl were uploaded into the Ingenuity Pathway Analysis (IPA) software in which the differentially regulated proteins were all characterized with statistical significance (*P* < 0.05)^22^. Based on the functional characterization in IPA, we showed three functional settings into which all the uploaded proteins were classified: molecular and cellular functions, physiological system development and functions, and hepatotoxicity. The differentially expressed proteins were shown to be involved in various biological processes (Supplementary Table S1), such as “Amino Acid Metabolism” and “Lipid Metabolism” (Fig. 3A), “Tissue Morphology” (Fig. 3B), and “Liver Steatosis” (Fig. 3C).

### Amino acid metabolism was altered by Leu deprivation in HepG2 cells

To test whether the metabolism of other amino acids was affected by Leu deprivation in HepG2 cells, we classified the amino acid metabolism-related differentially expressed proteins. We found amino acid metabolism was significantly altered in -Leu/Ctrl based on the function characterization of IPA analysis (Fig. 3A). A total of 11 differentially expressed proteins, such as betanine–homocysteine S-methyltransferase 1 (BHMT), and mitochondrial ornithine carbamoyltransferase (OTC), are involved in various amino acid metabolic pathways including cysteine and methionine metabolism, arginine and proline metabolism (Table 2).

**Table 2:** All the amino acid metabolism-related differentially expressed proteins.

Acession	Description	Gene Name	-Leu/Ctrl	Amino acid metabolism process
Q9H2A2	Aldehyde dehydrogenase family 8 member A1	*ALDH8A1*	11.577*	Val, Leu and Ile degradation; β-Ala metabolism; Arg and Pro metabolism; His metabolism; Lys degradation
P00439	Phenylalanine-4-hydroxylase	*PAH*	11.075*	Phe, Tyr and Trp biosynthesis
Q93088	Betaine–homocysteine S-methyltransferase 1	*BHMT*	7.652*	Gly, Ser and Thr metabolism; Cys and Met metabolism
P05166	Propionyl-CoA carboxylase beta chain, mitochondrial	*PCCB*	6.541*	Val, Leu and Ile degradation
Q9UI17	Dimethylglycine dehydrogenase, mitochondrial	*DMGDH*	5.834*	Gly, Ser and Thr metabolism
P30038	Delta-1-pyrroline-5-carboxylate dehydrogenase, mitochondrial	*ALDH4A1*	5.152*	Arg and Pro metabolism; Ala, Asp and Glu metabolism
P00480	Ornithine carbamoyltransferase, mitochondrial	*OTC*	4.747*	Arg and Pro metabolism
D4QEZ8	Short-chain acyl-CoA dehydrogenase	*ACADS*	4.739*	Val, Leu and Ile degradation
B2RBJ5	Alanine-glyoxylate aminotransferase 2	*AGXT2*	3.959*	Gly, Ser and Thr metabolism; Ala, Asp and Glu metabolism
P26440	Isovaleryl-CoA dehydrogenase, mitochondrial	*IVD*	2.722*	Val, Leu and Ile degradation
P42357	Histidine ammonia-lyase	*HAL*	2.559*	His metabolism

^*^ *P* < 0.05. Val, valine; Leu, leucine; Ile, isoleucine; Ala, alanine; Arg, arginine; Pro, proline; His, histidine; Lys, lysine; Phe, phenylalanine; Tyr, tyrosine; Trp, tryptophan; Gly, glycine; Ser, serine; Thr, threonine; Cys, cysteine; Met, methionine; Asp, aspartate; Glu, glutamate.

### Leu deprivation leads to the activation of fatty acid β-oxidation pathway in HepG2 cells

**Figure 4:**
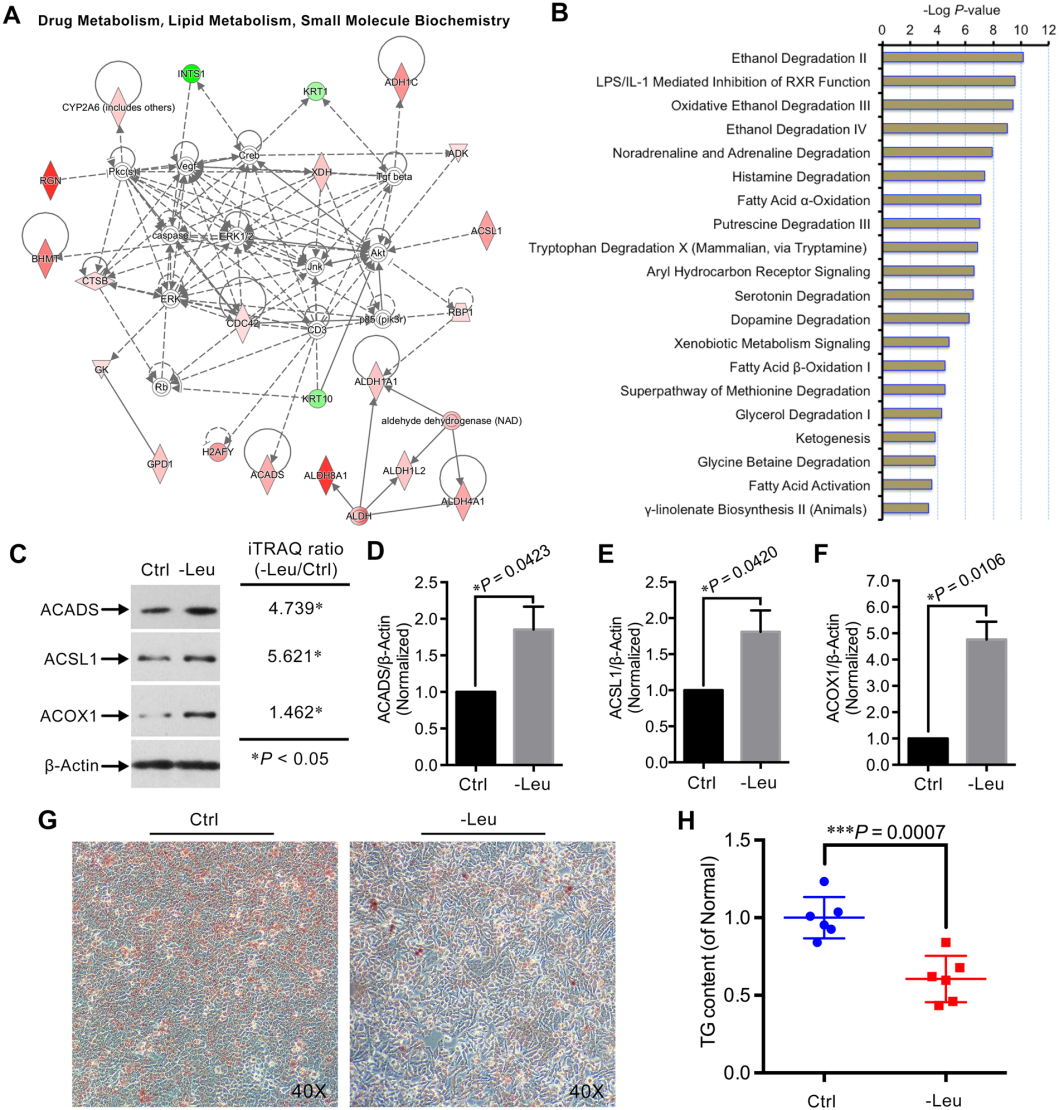
Leu deprivation activates the fatty acid β-oxidation pathway in HepG2 cells. (**A**) The most enriched interaction network of the differentially expressed proteins in Leu-deprived HepG2 cells. Red indicates up-regulated; green indicates down-regulated; white indicates proteins involved in certain network but not differentially expressed in this study. The degree of change for protein expression is indicated by color depth. (**B**) The 20 top canonical pathways ranked by the *P*-values in the IPA tools for the -Leu/Ctrl. (**C**) Immunoblotting of ACSL1, ACADS, ACOX1, and β-Actin in the HepG2 cells treated with Leu deprivation as indicated. (**D–F**) Quantitation of ACSL1/β-Actin, ACADS/β-Actin, and ACOX1/β-Actin as described in (**C**). Data are means ± SD (n = 3). ^*^ *P* < 0.05 (paired Student’s *t*-test). (**G**) HepG2 cells were cultured with normal and Leu-deprived media for 24 hours, then cells were stained with ORO and observed with a microscope at 40x magnification. Representative images were shown. (**H**) Cellular TG content was measured in HepG2 cells with different treatments as described in (**G**). Data are means ± SD (n = 6). ^***^ *P* < 0.001 (unpaired Student’s *t*-test).

Many proteins can only be effective through interacting with their partner(s)^23^, the exploration of interacting relationships among all the differentially expressed proteins is crucial to understand the integral biological role of relevant proteins upon certain treatment. Thus, we also performed the network analysis using the IPA tool, in which the interaction annotation is based on the microarray results of numerous publications^24^. Based on the scores, the network “Drug Metabolism, Lipid Metabolism, Small Molecule Biochemistry” was highly enriched in -Leu/Ctrl (Score 53; Fig. 4A). Combined with the functional characterization result (Fig. 3A), the interaction network analysis suggested the lipid metabolism might be altered by Leu deprivation in HepG2 cells. Interestingly, further analysis of the signaling pathways altered by Leu deprivation suggested several lipid metabolism-related metabolic pathways, particularly fatty acid metabolic pathways, including “Fatty Acid β-Oxidation I” (*P* = 3.74e^−05^), were significantly altered upon Leu deprivation (Fig. 4B). Two proteins long-chain-fatty-acid–CoA ligase 1 (ACSL1, 5.621-fold), and short-chain acyl-CoA dehydrogenase (ACADS, 4.739-fold), which are the key enzymes involved in the fatty acid β-oxidation pathway (Supplementary Fig. S3), were significantly up-regulated by Leu deprivation. In addition, the protein peroxisomal acyl-coenzyme A oxidase 1 (ACOX1), which also plays crucial role in the fatty acid β-oxidation, was likely up-regulated (1.462-fold) by Leu deprivation (Supplementary Fig. S3). To validate the LC-MS/MS data and confirm whether the fatty acid β-oxidation pathway was altered by Leu deprivation, we compared the ratios of ACSL1, ACADS, and ACOX1 between the Ctrl and -Leu by immunoblotting (Fig. 4C). The expressions of these three proteins were conformity with those determined by the iTRAQ labeled LC-MS/MS strategy (Fig. 4D–F). In addition, the cellular lipid content and accumulation were reduced by Leu deprivation in HepG2 cells (Fig. 4G,H). These results suggest that Leu deprivation leads to the activation of the fatty acid β-oxidation pathway in HepG2 cells.

## Discussion

In the present study, we for the first time comparatively characterized the proteome alteration induced by Leu deprivation in HepG2 cells using iTRAQ-coupled LC-MS/MS analysis. One of our major findings was that the fatty acid β-oxidation pathway was activated by Leu deprivation in HepG2 cells. Lipid metabolism, including fatty acid metabolism, is a primary function of the liver. In hepatocytes, excessive free fatty acids are incorporated into triglycerides that can then be stored in lipid droplets^25^, the lipid droplets will be degraded to produce fatty acids under nutrient deficiency conditions to maintain cellular energy homeostasis through the β-oxidation of fatty acids^25, 26^, a main energy-generating process that involves a series of proteins^27^. Among these proteins, ACSL1, which is highly expressed in liver^28^, is a vital enzyme that belongs to the class of acyl-CoA synthetases which are essential for the anabolism of lipid, and catabolism of fatty acids^29^. In hepatocytes, ACSL1 converts long-chain fatty acids into activated acyl-CoA, and the activated acyl-CoA then enters the β-oxidation pathway for producing energy^29, 30^. Decreases in ACSL1 activity significantly decreases triglyceride synthesis and fatty acid β-oxidation in hepatocytes or livers^31^. Additionally, ACADS, another crucial enzyme for the β-oxidation of fatty acids, catalyzes the first step of mitochondrial short-chain fatty acid β-oxidation^32^. Meanwhile, ACOX1 acts as the rate-limiting enzyme in the peroxisomal fatty acid β-oxidation pathway, another mechanism for fatty acid β-oxidation^33, 34^. In the present study, our iTRAQ data together with immunoblotting assays demonstrated that ACSL1, ACADS, and ACOX1 were highly up-regulated by Leu deprivation in HepG2 cells. Furthermore, we also found the cellular triglycerides were reduced by Leu deprivation in HepG2 cells, which might be partially caused by the activation of fatty acid β-oxidation pathway. Previously, a number of studies have demonstrated that Leu affects the content of cellular triglycerides in hepatocytes or livers. For instance, Guo and Cavener^10^ reported that dietary Leu deprivation down-regulated the fatty acid synthesis-related genes, thus leading to the reduction of triglycerides in mouse livers. In addition, Zarfeshani *et al*.^11^ recently showed the cellular triglycerides were accumulated upon 0.1 mM and 2.5 mM Leu supplementation in HepG2 cells. However, most of the related studies did not explore the connection between Leu deprivation/supplementation with the oxidation of fatty acids, which is also a main contributor for the cellular lipid content. Our finding thus suggests a new way for the regulation of lipid metabolism through the oxidation of fatty acids caused by Leu in hepatocytes. While the mechanism by which Leu deprivation activates hepatic fatty acid β-oxidation remains elusive, a possible explanation is that Leu deprivation might up-regulate several upstream transcription factors with transcriptional control over the lipid (fatty acid) metabolism genes. In support of this notion, our upstream analysis showed that the peroxisome proliferator-activated receptor (PPAR) family core members, PPAR-γ and PPAR-α, which both transcriptionally regulate *ACSL1* and *ACADS*, were predicated to be activated in Leu-deprived HepG2 cells (Supplementary Fig. S4).

In addition, our bioinformatics analysis showed that global amino acid metabolism was significantly altered by Leu deprivation (Fig. 3A). Also, we identified 11 differentially expressed proteins that are involved in amino acid metabolism (Table 2). Among these, the proteins aldehyde dehydrogenase family 8 member A1 (ALDH8A1), mitochondrial delta-1-pyrroline-5-carboxylate dehydrogenase (ALDH4A1), mitochondrial ornithine carbamoyltransferase (OTC), are involved in the metabolism of Arg. The protein OTC is a key involver of hepatic urea cycle pathway^35, 36^, in which Arg is a direct precursor for the production of urea^36^. The up-regulation of OTC suggested that the urea cycle might be activated by Leu deprivation, which was consistent with several previous studies^37, 38^. Interestingly, two controversial explanations were raised about the site of Leu affecting urea cycle: at glutamate dehydrogenase^39^ or at OTC^37^. Obviously, our result supported that the OTC is more likely the site since we found no differential expression of glutamate dehydrogenase after Leu deprivation in HepG2 cells. Together, our data suggested that Leu deprivation might promote the conversion of Arg into urea by up-regulating OTC, showing a potential way for Leu regulating Arg metabolism in HepG2 cells.

Also, our iTRAQ data together with pathway analysis have shown a number of crucial proteins were significantly regulated by Leu deprivation. The present study showed that two proteins ADH1C and ALDH1A1, which are the key enzymes in ethanol degradation pathways, were up-regulated by Leu deprivation in our iTRAQ data. Moreover, the differential expressions of these proteins were further validated by the immunoblotting assays. Once reaching to the cytosol of liver cells, which is the main site for the degradation of ethanol, ethanol will be converted to acetaldehyde by ADH^40^. The acetaldehyde, a highly toxic substance that causes multiple malfunctions in organism^41, 42^, will then be catalyzed into acetate by the ALDH^40, 43^. Previously, a number of studies have demonstrated that ethanol oxidation can be accelerated by specific amino acids. Tanaka *et al*.^44^ found that Ala could accelerate ethanol oxidation in the livers of rats treated with acute ethanol. And our previous research has found that the ethanol degradation pathways were activated upon the supplementation of another functional amino acid, Arg, in HepG2 cells^19^. Since the metabolism of amino acids can be affected by each other (also shown in this study), it is possible that, besides Ala and Arg, other amino acid(s) may also function in the ethanol degradation pathways. Our finding for the first time showed that Leu deprivation activated the ethanol degradation pathways, thus extended the idea that specific AAs (at least including Ala, Arg, and Leu) may have roles in ethanol metabolism in HepG2 cells. While needed to be further validated in other hepatocyte-derived cell lines as well as *in vivo* trials, this finding provides new insight into the role of Leu in attenuating the malfunction induced by acute or chronic exposure of ethanol in the liver.

In conclusion, by coupling iTRAQ with LC-MS/MS, we for the first time investigated the proteome response to Leu deprivation in HepG2 cells. Our data have shown that the cellular amino acid metabolism was significantly altered by the Leu treatment. Importantly, we found that Leu deprivation disturbed some biological processes that have not been connected with Leu treatments before, particularly the fatty acid β-oxidation pathway. Although this phenomenon needs to be further investigated in other liver cell lines as well as animal trials, our findings provide new insights into the regulation of fatty acid metabolism by specific amino acids, especially Leu in the HepG2 cell line.

## Materials and Methods

### Cell line and culture

The human hepatocellular carcinoma (HepG2) cell line, which is a hepatocyte-derived cell line and has been widely used as an *in vitro* hepatocyte model (e.g., see refs 45–47), was a kind gift from Dr. Zaiqing Yang (Huazhong Agricultural University, College of Life Science and Technology). HepG2 cells were grown in RPMI-1640 (11875, Gibco) supplemented with 10% fetal bovine serum (1660516, Gibco) and 1% penicillin-streptomycin (15070, Invitrogen). To produce the Leu-deprived medium, RPMI-1640 without leucine, arginine, and lysine (R1780, Sigma-Aldrich) was supplemented with 200 mg/L (final concentration) arginine (0953, Amresco), 40 mg/L (final concentration) lysine (M234, Amresco), 10% fetal bovine serum and 1% penicillin-streptomycin. When cells were grown to approximately 80% confluence, twelve dishes of cells were randomly divided into two groups treated as follows: 1) for normal group (Ctrl), six dishes of HepG2 cells were cultured in fresh complete medium for 50 min; 2) for Leu deprivation group (-Leu), six dishes of HepG2 cells were cultured in Leu-deprived medium for 50 min. All the cell cultures were performed at 37 °C under 5% CO_2_. For iTRAQ experiments, two independent biological replicates were performed to increase the statistical confidence.

### Protein preparation and digestion, iTRAQ labeling

Six dishes of harvested HepG2 cells for each group were pooled into one sample to reduce the individual error as previously described^19^. The pooled cells were then lysed with the buffer (7 M Urea, 2 M Thiourea, 4% CHAPS, 40 mM Tris-HCl, pH 8.5, 1 mM PMSF, 2 mM EDTA) and sonicated in ice. The total proteins were reduced with 10 mM DTT at 56 °C for 1 h, alkylated with 55 mM IAM in the darkroom for 1 h, and precipitated by adding 4 × volume of chilled acetone at −20 °C overnight. Then the samples were centrifuged at 4 °C, 30,000 g. The pellet was dissolved in 0.5 M TEAB (Applied Biosystems, Milan, Italy) and sonicated in ice. After centrifuged at 4 °C, 30,000 g, the protein concentration of supernatant was determined of by Bradford essay. Approximately 100 μg of total protein was digested with trypsin to protein ratio of 30:1 at 37 °C for 16 h. The digested peptides were dried by vacuum centrifugation, reconstituted in 0.5 M TEAB and processed according to the manufacture’s protocol (Applied Biosystems). Samples were labeled with the iTRAQ reagents below: Ctrl (tag 113) and -Leu (tag 114). The labeled peptides were then incubated at room temperature for 2 h and vacuum-dried.

### Strong cation exchange (SCX) chromatography

SCX chromatography was carried out to remove all interfering substances with an LC-20AB HPLC Pump system (Shimadzu, Kyoto, Japan). The labeled peptide mixtures were reconstituted with 4 mL buffer A (25 mM NaH_2_PO_4_ in 25% acetonitrile, pH 2.7) and loaded onto a 4.6 × 250 mm Ultremex SCX column containing 5-μm particles (Phenomenex). The peptides were eluted at a flow rate of 1 mL/min with a gradient of buffer A for 10 min, 5–60% buffer B (25 mM NaH_2_PO_4_, 1 M KCl in 25% acetonitrile, pH 2.7) for 27 min, 60–100% buffer B for 1 min, and then maintained at 100% buffer B for 1 min, equilibrated with buffer A for 10 min. The eluted peptides were collected every 1 min and pooled into 20 fractions, desalted using a Strata X C18 column (Phenomenex) and vacuum-dried.

### LC-ESI-MS/MS analysis based on Q EXACTIVE

The Q EXACTIVE based LC-ESI-MS/MS was performed as previously described with minor modifications^48^. Fractions were re-suspended in solvent A (2% acetonitrile, 0.1% formic acid) and centrifuged at 20,000 g for 10 min. Then, 5 μg of peptide mixture was loaded onto a 2 cm C18 trap column on an LC-20AD nanoHPLC (Shimadzu, Kyoto, Japan), and then eluted onto a 10 cm analytical C18 column (inner diameter 75 μm). The samples were loaded at 8 μL/min for 4 min, then the gradients run: 300 nL/min for 44 min in 2–35% solvent B (98% acetonitrile, 0.1% formic acid), linear gradient to 80% solvent B for 2 min, and maintained in 80% solvent B for 4 min, and finally returned to 5% in 1 min. Tandem mass spectrometry (MS/MS) was performed with Q EXACTIVE (Thermo Fisher Scientific, San Jose, CA) coupled online to the HPLC, following nanoelectrospray ionization. Intact peptides were detected in the Orbitrap at a resolution of 70,000. Peptides were selected for MS/MS using high-energy collision dissociation (HCD) operating mode with a normalized collision energy setting of 27.0 (±12%); ion fragments were detected in the Orbitrap at a resolution of 17,500. A data-dependent procedure that alternated between one MS scan followed by 15 MS/MS scans was applied for the 15 most abundant precursor ions above a threshold ion count of 20,000 in the MS survey scan with a following Dynamic Exclusion duration of 15 s. The electrospray voltage was set to 1.6 kV. Automatic gain control (AGC) was used to optimize the spectra generated by the Orbitrap and the AGC target for full MS was 3e6 and 1e5 for MS/MS. The *m/z* scan range of 350–2,000 Da for MS scan, and 100–1,800 Da for MS/MS scan.

### Bioinformatics analysis

Raw data files generated from the LC-MS/MS were converted into MGF files using 5600 msconverter, and the MGF files were searched. Proteins were identified using Mascot search engine (Matrix Science, London, UK; version 2.3.02) against Uniprot homo database. The search criteria for protein identification were a mass tolerance of 20 ppm for intact peptide masses, and 0.05 Da for fragmented ions. Only one missed cleavage was allowed. Gln → pyro-Glu (N-term Q), oxidation (M), deamidated (NQ) and carbamidomethyl (C), iTRAQ8plex (N-term), iTRAQ8plex (K) were chosen as the potential variable modifications and fixed modifications, respectively. The charge states of peptides were set to +2 and +3. Specifically, an automatic decoy database search was performed in Mascot by choosing the decoy checkbox in which a random sequence of database was generated and tested for raw spectra as well as the real database^49^. To reduce false positive results, all data were reported based on a 95% confidence and false discovery rate (FDR) less than 1%. All the confident protein identifications involve at least one unique peptide. The quantitative protein ratios were weighted and normalized by the median ratio in Mascot. All the results of quantitative proteins were then exported into Microsoft Excel software (14.4.9) for manual data interpretation. To fully identify the proteins regulated by Leu deprivation, we used two kinds of criteria to consider the differential expressions of proteins as follows: 1) for the proteins quantified in both the two sets of iTRAQ experiments, the differentially expressed proteins were defined by criteria of unique peptide >1, *P*-value < 0.05, fold change >1.2 (or <0.833) in both the two iTRAQ experiments^50–52^; 2) for the proteins quantified in only one set of iTRAQ experiment, the differentially expressed proteins were defined by criteria of unique peptide >1, *P*-value < 0.05, fold change >2 (or <0.5) in any one set of the two iTRAQ experiments^19, 21^.

Gene Ontology (GO) analysis was performed using Blast2GO program. The Kyoto Encyclopedia of Genes and Genomes (KEGG) database (http://www.genome.jp/kegg/) was used to study the amino acid metabolism and display the detail of fatty acid degradation pathway. Functional characterization, interaction network and pathway analysis were conducted using Ingenuity Pathway Analysis (IPA) software (www.ingenuity.com).

### Immunoblotting assays

HepG2 cells were lysed using lysis buffer (50 mM Tris, 150 mM NaCl, 1 mM EDTA, 1% Triton X-100) supplemented with protease inhibitors (1 μg/mL Leupeptin, 1 μg/mL Aprotinin, 1 μg/mL Pepstatin, and 50 μg/mL PMSF). Equal amounts of cell lysates were resolved by 10% SDS-PAGE. Proteins were then transferred onto PVDF membranes (16916600, Roche), blocked with 5% non-fat milk in Tris-buffered saline containing 0.05% Tween 20 for 1 hour, and probed with primary antibodies against ADH1C (A8081, ABclonal Technology), ALDH1A1 (A1802, ABclonal Technology), ACSL1 (A1000, ABclonal Technology), ACOX1 (A8091, ABclonal Technology), ACADS (A0945, ABclonal Technology), and β-Actin (4967L, Cell Signaling Technology). The specific proteins were detected with HRP-conjugated secondary antibodies (sc-2004, Santa Cruz Biotechnology), developed with SuperSignal West Pico Chemiluminescent Substrate (34080, Thermo Scientific) and visualized by Kodak Image Station 2000 MM. Immunoblotting results were quantified using Image J software (1.49s). All the immunoblotting assays were performed in three biological replicates.

### Lipid accumulation and content measurements

Cellular lipid accumulation quantification was performed by Oil Red O (ORO) staining. In detail, HepG2 cells were cultured overnight in 6-well culture plates. Cell media were then replaced with fresh normal and Leu-deprived media, respectively. Cells were cultured for 24 h at 37 °C under 5% CO_2_. Cells were then fixed in 4% paraformaldehyde for 30 min, washed three times with PBS and stained with pre-warmed diluted ORO solution (O1391, Sigma-Aldrich) for 1 h in darkness. The droplets were observed with light microscope (CKX31, Olympus). Cellular TG content was measured with the Triglyceride Assay Kit (A110-1, Nanjing Jiancheng Bioengineering Institute, China) following the manufacturer’s instructions. The measurements were performed in six biological replicates.

### Statistical analysis

All statistical analyses for immunoblotting assays and the lipid accumulation and content measurements were performed using GraphPad Prism software (6.0c). Values are shown as mean ± standard deviation (SD), and statistical significance was calculated using two-tailed Student’s *t*-test. Differences were considered statistically significant at *P* < 0.05.

## Electronic supplementary material

Supplementary Information

## References

[1] Lynch CJ (2006). Leucine in food mediates some of the postprandial rise in plasma leptin concentrations. Am J Physiol Endocrinol Metab.

[2] Blouet C, Schwartz GJ (2012). Brainstem nutrient sensing in the nucleus of the solitary tract inhibits feeding. Cell Metab.

[3] Yan X (2012). Reconstitution of leucine-mediated autophagy via the mTORC1-Barkor pathway *in vitro*. Autophagy.

[4] Wu H (2012). MiR-20a and miR-106b negatively regulate autophagy induced by leucine deprivation via suppression of ULK1 expression in C2C12 myoblasts. Cell Signal.

[5] Yang J, Chi Y, Burkhardt BR, Guan Y, Wolf BA (2010). Leucine metabolism in regulation of insulin secretion from pancreatic beta cells. Nutr Rev.

[6] Pedroso JA, Zampieri TT, Donato J (2015). Reviewing the Effects of L-Leucine Supplementation in the Regulation of Food Intake, Energy Balance, and Glucose Homeostasis. Nutrients.

[7] Sun X, Zemel MB (2007). Leucine and calcium regulate fat metabolism and energy partitioning in murine adipocytes and muscle cells. Lipids.

[8] Sun X, Zemel MB (2009). Leucine modulation of mitochondrial mass and oxygen consumption in skeletal muscle cells and adipocytes. Nutr Metab (Lond).

[9] Hutson SM, Wallin R, Hall TR (1992). Identification of mitochondrial branched chain aminotransferase and its isoforms in rat tissues. J Biol Chem.

[10] Guo F, Cavener DR (2007). The GCN2 eIF2alpha kinase regulates fatty-acid homeostasis in the liver during deprivation of an essential amino acid. Cell Metab.

[11] Zarfeshani A, Ngo S, Sheppard AM (2014). Leucine alters hepatic glucose/lipid homeostasis via the myostatin-AMP-activated protein kinase pathway - potential implications for nonalcoholic fatty liver disease. Clin Epigenetics.

[12] Lenaerts K (2007). Arginine deficiency in preconfluent intestinal Caco-2 cells modulates expression of proteins involved in proliferation, apoptosis, and heat shock response. Proteomics.

[13] Lenaerts K, Mariman E, Bouwman F, Renes J (2006). Glutamine regulates the expression of proteins with a potential health-promoting effect in human intestinal Caco-2 cells. Proteomics.

[14] Deniel N (2007). Glutamine regulates the human epithelial intestinal HCT-8 cell proteome under apoptotic conditions. Mol Cell Proteomics.

[15] Xin L (2007). Applying proteomic methodologies to analyze the effect of methionine restriction on proliferation of human gastric cancer SGC7901 cells. Clin Chim Acta.

[16] Sun H (2012). iTRAQ-coupled 2D LC-MS/MS analysis on differentially expressed proteins in denervated tibialis anterior muscle of Rattus norvegicus. Mol Cell Biochem.

[17] Yan G, Yan X (2015). Ribosomal proteomics: Strategies, approaches, and perspectives. Biochimie.

[18] Treumann A, Thiede B (2010). Isobaric protein and peptide quantification: perspectives and issues. Expert Rev Proteomics.

[19] Yan G (2016). Comparative Proteomics Analysis Reveals L-Arginine Activates Ethanol Degradation Pathways in HepG2 Cells. Sci Rep.

[20] Long B (2016). Quantitative proteomics analysis reveals glutamine deprivation activates fatty acid beta-oxidation pathway in HepG2 cells. Amino Acids.

[21] Mancias JD, Wang X, Gygi SP, Harper JW, Kimmelman AC (2014). Quantitative proteomics identifies NCOA4 as the cargo receptor mediating ferritinophagy. Nature.

[22] An K (2014). Quantitative proteomic analysis reveals that transmissible gastroenteritis virus activates the JAK-STAT1 signaling pathway. J Proteome Res.

[23] Alberts B (1998). The cell as a collection of protein machines: preparing the next generation of molecular biologists. Cell.

[24] Huang J (2014). iTRAQ-proteomics and bioinformatics analyses of mammary tissue from cows with clinical mastitis due to natural infection with Staphylococci aureus. BMC Genomics.

[25] Cabodevilla AG (2013). Cell survival during complete nutrient deprivation depends on lipid droplet-fueled beta-oxidation of fatty acids. J Biol Chem.

[26] Dong H, Czaja MJ (2011). Regulation of lipid droplets by autophagy. Trends Endocrinol Metab.

[27] Houten SM, Wanders RJ (2010). A general introduction to the biochemistry of mitochondrial fatty acid beta-oxidation. J Inherit Metab Dis.

[28] Mashek DG, Li LO, Coleman RA (2006). Rat long-chain acyl-CoA synthetase mRNA, protein, and activity vary in tissue distribution and in response to diet. J Lipid Res.

[29] Zhao ZD (2016). Characterization of the promoter region of the bovine long-chain acyl-CoA synthetase 1 gene: Roles of E2F1, Sp1, KLF15, and E2F4. Sci Rep.

[30] Hall AM, Smith AJ, Bernlohr DA (2003). Characterization of the Acyl-CoA synthetase activity of purified murine fatty acid transport protein 1. J Biol Chem.

[31] Li LO (2009). Liver-specific loss of long chain acyl-CoA synthetase-1 decreases triacylglycerol synthesis and beta-oxidation and alters phospholipid fatty acid composition. J Biol Chem.

[32] van Maldegem BT (2006). Clinical, biochemical, and genetic heterogeneity in short-chain acyl-coenzyme A dehydrogenase deficiency. JAMA.

[33] Baarine M (2012). Evidence of oxidative stress in very long chain fatty acid–treated oligodendrocytes and potentialization of ROS production using RNA interference-directed knockdown of ABCD1 and ACOX1 peroxisomal proteins. Neuroscience.

[34] Nam KW (2013). Tert-butylhydroquinone reduces lipid accumulation in C57BL/6 mice with lower body weight gain. Arch Pharm Res.

[35] Gautier C, Husson A, Vaillant R (1976). [Effects of glucocorticosteroids on enzymatic activity in the urea cycle in fetal rat liver]. Biochimie.

[36] Morris SM (2002). Regulation of enzymes of the urea cycle and arginine metabolism. Annu Rev Nutr.

[37] Rognstad R (1977). Sources of ammonia for urea synthesis in isolated rat liver cells. Biochim Biophys Acta.

[38] McGivan JD, Bradford NM, Crompton M, Chappell JB (1973). Effect of L-leucine on the nitrogen metabolism of isolated rat liver mitochondria. Biochem J.

[39] Mourao JM, McGivan JD, Chappell JB (1975). The effects L-leucine on the synthesis of urea, glutamate and glutamine by isolated rat liver cells. Biochem J.

[40] Lieber CS (1991). Hepatic, metabolic and toxic effects of ethanol: 1991 update. Alcohol Clin Exp Res.

[41] Jackson B (2011). Update on the aldehyde dehydrogenase gene (ALDH) superfamily. Hum Genomics.

[42] Quertemont E, Tambour S (2004). Is ethanol a pro-drug? The role of acetaldehyde in the central effects of ethanol. Trends Pharmacol Sci.

[43] Vasiliou V, Pappa A, Estey T (2004). Role of human aldehyde dehydrogenases in endobiotic and xenobiotic metabolism. Drug Metab Rev.

[44] Tanaka T (1993). Effects of alanine and glutamine administration on the inhibition of liver regeneration by acute ethanol treatment. Alcohol Alcohol Suppl.

[45] Song YM (2012). Dimethyl sulfoxide reduces hepatocellular lipid accumulation through autophagy induction. Autophagy.

[46] Bhatia H, Pattnaik BR, Datta M (2016). Inhibition of mitochondrial beta-oxidation by miR-107 promotes hepatic lipid accumulation and impairs glucose tolerance *in vivo*. Int J Obes (Lond).

[47] Martinez LO (2003). Ectopic beta-chain of ATP synthase is an apolipoprotein A-I receptor in hepatic HDL endocytosis. Nature.

[48] Xie L (2015). Proteome-wide lysine acetylation profiling of the human pathogen Mycobacterium tuberculosis. Int J Biochem Cell Biol.

[49] Zeng S (2015). Proteome analysis of porcine epidemic diarrhea virus (PEDV)-infected Vero cells. Proteomics.

[50] Ren Y (2013). Hypoxia modulates A431 cellular pathways association to tumor radioresistance and enhanced migration revealed by comprehensive proteomic and functional studies. Mol Cell Proteomics.

[51] Huang HY (2015). RhoGDIbeta Inhibits Bone Morphogenetic Protein 4 (BMP4)-induced Adipocyte Lineage Commitment and Favors Smooth Muscle-like Cell Differentiation. J Biol Chem.

[52] Shi Q (2015). Proteomics analyses for the global proteins in the brain tissues of different human prion diseases. Mol Cell Proteomics.

