# Supplementary material for: The Fatty Acid β-Oxidation Pathway is Activated by Leucine Deprivation in HepG2 Cells: A Comparative Proteomics Study
Source: Sci Rep. 2017 May 15;7:1914. doi: 10.1038/s41598-017-02131-2 (PMC5432498; doi:10.1038/s41598-017-02131-2)
Supplement: Supplementary file 1 — Supplementary Information [file 41598_2017_2131_MOESM1_ESM.pdf]

Supplementary Information:

The Fatty Acid β-Oxidation Pathway is Activated by Leucine Deprivation in HepG2 Cells: A Comparative Proteomics Study

Guokai Yan[1,2,3], Xiuzhi Li[1,2,3], Ying Peng[2,4], Baisheng Long[1,2,3], Qiwen Fan[1,2,3], Zhichang Wang[1,2,3], Min Shi[1,2,3], Chunlin Xie[1,2,3], Li Zhao[1,2,3] & Xianghua Yan[1,2,3]

[1]College of Animal Sciences and Technology, Huazhong Agricultural University, Wuhan, 430070, Hubei, China

[2]The Cooperative Innovation Center for Sustainable Pig Production, Wuhan, 430070, Hubei, China

[3]Hubei Provincial Engineering Laboratory for Pig Precision Feeding and Feed Safety, Wuhan, 430070, Hubei, China

[4]State Key Laboratory of Agricultural Microbiology, College of Veterinary Medicine, Huazhong Agricultural University, Wuhan, 430070, Hubei, China

Correspondence and requests for materials should be addressed to X.Y. (email: xhyan@mail.hzau.edu.cn)

Content:

Supplementary Figure S1: Page 2

Supplementary Figure S2: Page 3

Supplementary Figure S3: Page 4

Supplementary Figure S4: Page 5

Supplementary Table S1: Pages 6-7

Supplementary Figure S1:

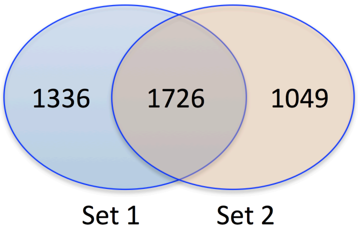

Supplementary Figure S1. Venn diagram of the number of quantified proteins derived from the two sets of iTRAQ experiments.

Supplementary Figure S2:

-Leu/Ctrl, Set1 vs Set2:

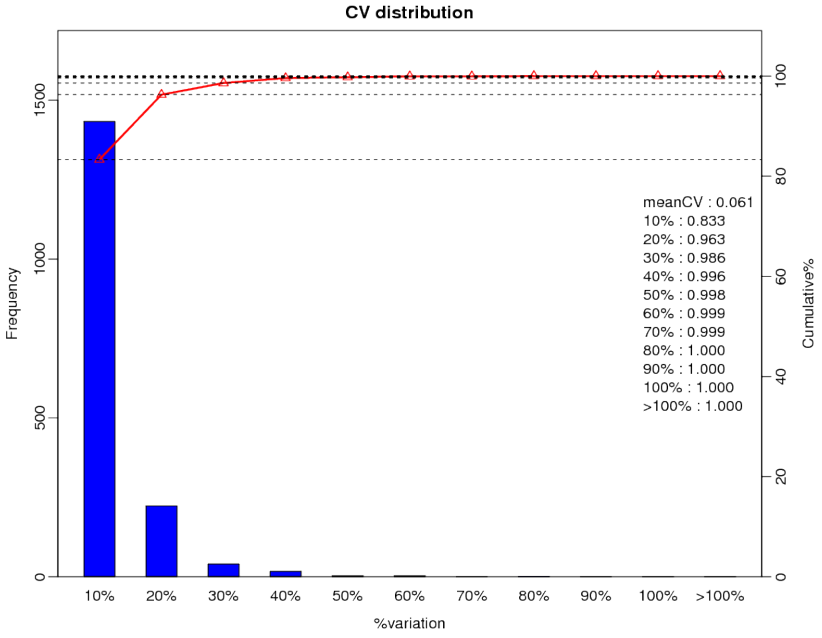

Supplementary Figure S2. The variation between the two iTRAQ runs.

Supplementary Figure S3:

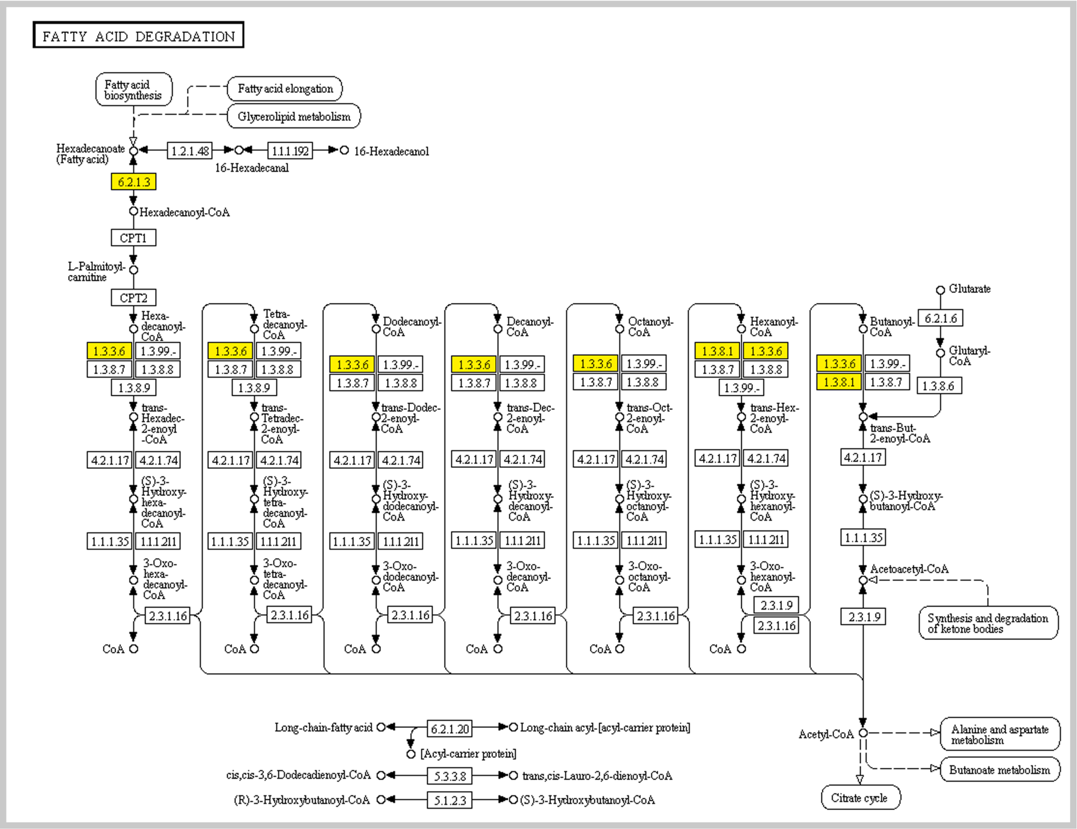

	6.2.1.3	1.3.8.1	1.3.3.6
Protein	ACSL1	ACADS	ACOX1
-Leu/Ctrl	5.621*	4.739*	1.462*

Supplementary Figure S3. The information of “Fatty Acid Degradation” pathway from the KEGG database. Yellow, the proteins differentially regulated by Leu deprivation.

Supplementary Figure S4:

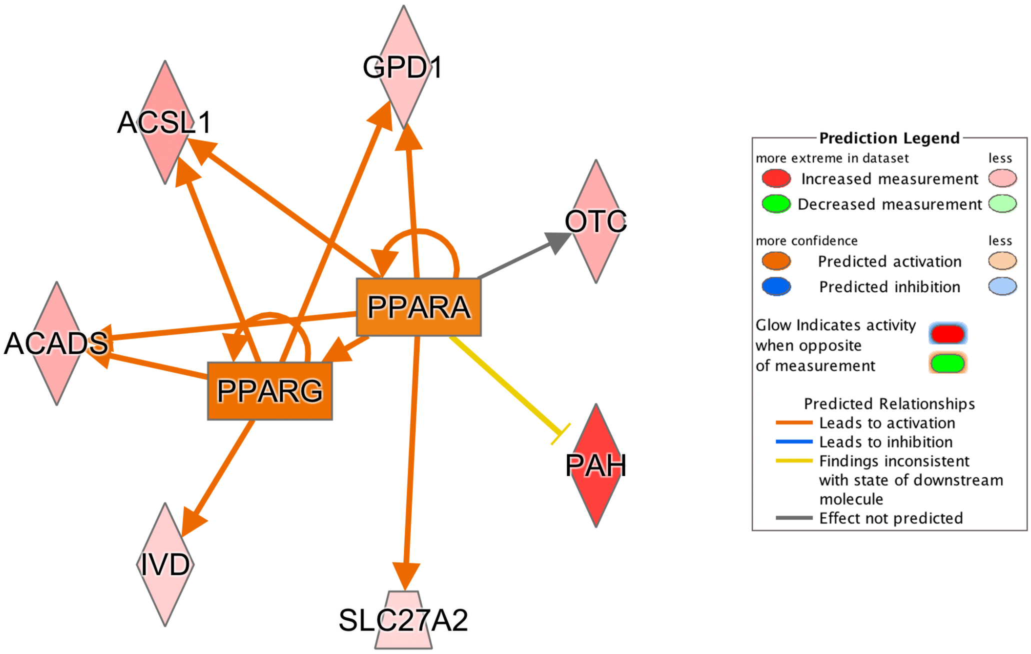

Supplementary Figure S4. Representative upstream analysis results of the differentially expressed proteins in Leu-deprived HepG2 cells using the IPA tools.

**Supplementary Table S1. The details of functional characterization of the differentially expressed proteins for -Leu/Ctrl.**			
**Molecular and cellular function**			
**Category**	**p-value**	**Molecules**	**# Molecules**
Amino Acid Metabolism	1.59E-10	AGXT2, BHMT, DMGDH, HAL, IVD, OTC, PAH, PCCB, QDPR, RGN	10
Small Molecule Biochemistry	1.59E-10	ACADS, ACSL1, ADH1C, ADK, ALDH1A1, ALDH8A1, AGXT2, BHMT, CDC42, CTSB, CYP2A6 (includes others), CYP2D6, DMGDH, GK, GPD1, H2AFY, HAL, HBB, IVD, KRT1, NPC2, OTC, PAH, PCCB, QDPR, RBP1, RGN, SLC27A2, STARD10, XDH	30
Drug Metabolism	6.25E-09	ACSL1, ADH1C, ALDH1A1, ALDH8A1, CTSB, CYP2A6 (includes others), CYP2D6, RBP1, XDH	9
Lipid Metabolism	6.25E-09	ACADS, ACSL1, ADH1C, ALDH1A1, ALDH8A1, BHMT, CDC42, CTSB, CYP2A6 (includes others), CYP2D6, GK, H2AFY, HBB, NPC2, PCCB, RBP1, RGN, SLC27A2, STARD10, XDH	20
Vitamin and Mineral Metabolism	6.25E-09	ADH1C, ALDH1A1, ALDH8A1, CYP2A6 (includes others), CYP2D6, KRT1, NPC2, RBP1, RGN, SLC27A2, XDH	11
Molecular Transport	1.28E-07	ACADS, ACSL1, ADH1C, ADK, ALDH1A1, ALDH8A1, BHMT, CDC42, CTSB, CYP2D6, H2AFY, HBB, NPC2, PAH, RBP1, RGN, SLC27A2, STARD10, XDH	19
Energy Production	7.06E-07	ACADS, ACSL1, ADH1C, ALDH1A1, CYP2D6, GPD1, HBB, RBP1, SLC27A2, XDH	10
Nucleic Acid Metabolism	1.02E-05	ADK, BHMT, CDC42, CYP2A6 (includes others), CYP2D6, GNB2, GPD1, XDH	8
Cell-To-Cell Signaling and Interaction	2.42E-05	CDC42, CTSB, CYP2D6, NPC2, XDH	5
Cell Death and Survival	1.27E-04	ADK, CTSB, HBB, KRT10, NPC2, WDR81, XDH	7
Cellular Compromise	1.27E-04	ACSL1, CDC42, CTSB, CYP2D6, NPC2, WDR81, XDH	7
RNA Post-Transcriptional Modification	1.80E-04	CYP2D6, XDH	2
Free Radical Scavenging	8.16E-04	CDC42, CYP2A6 (includes others), CYP2D6, HBB, XDH	5
Carbohydrate Metabolism	1.27E-03	ACSL1, ALDH1A1, BHMT, CDC42, CTSB, GK, GPD1, RBP1, RGN, XDH	10
Cellular Growth and Proliferation	1.28E-03	ALDH1A1, BHMT, CDC42, CTSB, KRT10, TIMELESS, XDH	7
Cell Morphology	2.04E-03	CDC42, CTSB, KRT1, NPC2, XDH	5
Cellular Assembly and Organization	2.04E-03	CDC42, CTSB, KRT1, XDH	4
Cellular Development	2.04E-03	ALDH1A1, BHMT, CDC42, CTSB, KRT10, NPC2, TIMELESS, XDH	8
Cellular Function and Maintenance	2.04E-03	CDC42, XDH	2
Cellular Movement	2.04E-03	CDC42, CTSB, RGN, XDH	4
DNA Replication, Recombination, and Repair	2.04E-03	ADK, CDC42, GPD1	3
Immune Cell Trafficking	2.04E-03	CDC42	1
Protein Synthesis	2.75E-03	ACADS, ALDH1A1, CDC42, CTSB, HBB, IVD, RIDA	7
Cell Cycle	4.07E-03	CDC42	1
Gene Expression	4.07E-03	RIDA	1
Post-Translational Modification	4.07E-03	ACADS, ALDH1A1, CDC42, CTSB, OTC, XDH	6
Cell Signaling	8.12E-03	CDC42, OTC, XDH	3
Protein Trafficking	8.12E-03	CDC42	1
**Physiological system development and function**			
**Category**	**p-value**	**Molecules**	**# Molecules**
Tissue Morphology	1.27E-04	ACSL1, ALDH1A1, BHMT, CDC42, CTSB, NPC2, WDR81, XDH	8
Embryonic Development	9.83E-04	ALDH1A1, BHMT, CDC42, HBB, NPC2, RBP1, RGN, TIMELESS	8
Organ Development	9.83E-04	ADK, ALDH1A1, CTSB, HBB, NPC2, RBP1, RGN, TIMELESS, XDH	9
Organismal Development	9.83E-04	ALDH1A1, BHMT, CDC42, CTSB, H2AFY, HBB, NPC2, RBP1, RGN, STARD10, TIMELESS, XDH	12
Respiratory System Development and Function	9.83E-04	CDC42, HBB, NPC2, RBP1, RGN, TIMELESS	6

Tissue Development	9.83E-04	ALDH1A1, CDC42, CTSB, HBB, KRT10, NPC2, RBP1, RGN, RIDA, TIMELESS, XDH	11
Cardiovascular System Development and Function	2.04E-03	CDC42, CTSB, XDH	3
Nervous System Development and Function	2.04E-03	ALDH1A1, CDC42	2
Tumor Morphology	2.04E-03	CTSB, RGN	2
Connective Tissue Development and Function	2.44E-03	ALDH1A1, CDC42, CTSB, NPC2, XDH	5
Inflammatory Response	3.70E-03	ADK, CTSB, GPD1, HBB, KRT1, KRT10, NPC2, RIDA, XDH	9
Digestive System Development and Function	4.07E-03	ADK, BHMT, CDC42, CTSB, RBP1, RGN, STARD10, XDH	8
Hepatic System Development and Function	4.07E-03	ADK, BHMT, CTSB, RBP1, RGN, STARD10, XDH	7
Organ Morphology	4.07E-03	ALDH1A1, BHMT, CTSB, H2AFY, HBB, KRT1, NPC2, RBP1, RGN, STARD10	10
Reproductive System Development and Function	4.07E-03	CDC42	1
Skeletal and Muscular System Development and Function	4.07E-03	ALDH1A1, CDC42, CTSB	3
Visual System Development and Function	4.07E-03	ALDH1A1, RBP1	2
Renal and Urological System Development and Function	6.09E-03	BHMT, CDC42, TIMELESS, XDH	4
Endocrine System Development and Function	8.12E-03	CTSB	1
Humoral Immune Response	1.22E-02	CTSB	1
Behavior	1.42E-02	CYP2A6 (includes others)	1
Hair and Skin Development and Function	1.42E-02	CTSB, KRT1	2
Hematological System Development and Function	1.62E-02	NPC2	1

Hepatotoxicity

**Category**	**p-value**	**Molecules**	**# Molecules**
Liver Steatosis	6.69E-07	ACADS, ACSL1, ADK, BHMT, GPD1, H2AFY, RGN, STARD10	8
Liver Cholestasis	6.24E-06	ACSL1, ADH1C, GK, PAH	4
Hepatocellular Carcinoma	2.29E-03	AGXT2, ALDH8A1, BHMT, H2AFY, HAL, HBB, RGN	7
Liver Hyperplasia/Hyperproliferation	2.29E-03	ACSL1, AGXT2, ALDH1A1, ALDH4A1, ALDH8A1, BHMT, CNOT1, CTSB, CYP2A6 (includes others), CYP2D6, H2AFY, HAL, HBB, INTS1, IVD, KRT10, PAH, RGN	18
Liver Fibrosis	4.07E-03	ADK, CTSB, XDH	3
Liver Proliferation	4.07E-03	CTSB, XDH	2
Liver Damage	1.22E-02	ADK, ALDH1A1, CTSB, RGN	4
Liver Inflammation/Hepatitis	1.22E-02	ADK	1
Liver Necrosis/Cell Death	3.65E-02	CTSB, RGN	2
Liver Cirrhosis	8.09E-02	ADK, PAH	2
